# Design and Development of a New Type of Hybrid PLGA/Lipid Nanoparticle as an Ursolic Acid Delivery System against Pancreatic Ductal Adenocarcinoma Cells

**DOI:** 10.3390/ijms23105536

**Published:** 2022-05-16

**Authors:** Adam Markowski, Anna Jaromin, Paweł Migdał, Ewa Olczak, Adrianna Zygmunt, Magdalena Zaremba-Czogalla, Krzysztof Pawlik, Jerzy Gubernator

**Affiliations:** 1 Department of Lipids and Liposomes, Faculty of Biotechnology, University of Wrocław, Joliot-Curie 14a, 50-383 Wrocław, Poland; anna.jaromin@uwr.edu.pl (A.J.); 298425@uwr.edu.pl (E.O.); adrianna.zygmunt@uwr.edu.pl (A.Z.); magdalena.zaremba-czogalla@uwr.edu.pl (M.Z.-C.); 2 Polish Academy of Science Ludwik Hirszfeld Institute of Immunology and Experimental Therapy, Weigla 12, 53-114 Wrocław, Poland; pawel.migdal@hirszfeld.pl (P.M.); krzysztof.pawlik@hirszfeld.pl (K.P.); 3 Department of Environment Hygiene and Animal Welfare, Bee Division, Wroclaw University of Environmental and Life Sciences, Chelmońskiego 38C, 51-630 Wrocław, Poland

**Keywords:** PLGA, pancreatic cancer, nanotechnology, cancer, nanoparticles, ursolic acid, terpenoids

## Abstract

Despite many attempts, trials, and treatment procedures, pancreatic ductal adenocarcinoma (PDAC) still ranks among the most deadly and treatment-resistant types of cancer. Hence, there is still an urgent need to develop new molecules, drugs, and therapeutic methods against PDAC. Naturally derived compounds, such as pentacyclic terpenoids, have gained attention because of their high cytotoxic activity toward pancreatic cancer cells. Ursolic acid (UA), as an example, possesses a wide anticancer activity spectrum and can potentially be a good candidate for anti-PDAC therapy. However, due to its minimal water solubility, it is necessary to prepare an optimal nano-sized vehicle to overcome the low bioavailability issue. Poly(lactic-co-glycolic acid) (PLGA) polymeric nanocarriers seem to be an essential tool for ursolic acid delivery and can overcome the lack of biological activity observed after being incorporated within liposomes. PLGA modification, with the addition of PEGylated phospholipids forming the lipid shell around the polymeric core, can provide additional beneficial properties to the designed nanocarrier. We prepared UA-loaded hybrid PLGA/lipid nanoparticles using a nanoprecipitation method and subsequently performed an MTT cytotoxicity assay for AsPC-1 and BxPC-3 cells and determined the hemolytic effect on human erythrocytes with transmission electron microscopic (TEM) visualization of the nanoparticles and their cellular uptake. Hybrid UA-loaded lipid nanoparticles were also examined in terms of their stability, coating dynamics, and ursolic acid loading. We established innovative and repeatable preparation procedures for novel hybrid nanoparticles and obtained biologically active nanocarriers for ursolic acid with an IC50 below 20 µM, with an appropriate size for intravenous dosage (around 150 nm), high homogeneity of the sample (below 0.2), satisfactory encapsulation efficiency (up to 70%) and excellent stability. The new type of hybrid UA-PLGA nanoparticles represents a further step in the development of potentially effective PDAC therapies based on novel, biologically active, and promising triterpenoids.

## 1. Introduction

Despite all efforts, pancreatic ductal adenocarcinoma remains one of the deadliest types of cancers to be diagnosed with, due to its high chemoresistance, high and early metastatic potential, and low diagnosis efficiency in the early stages of disease progression, where PDAC patients can fully recover with surgical methods [1,2,3]. Most of the commonly used chemotherapies, based on gemcitabine or, in a smaller number of cases, FOLFIRINOX (the combination of folinic acid, 5-fluorouracil, irinotecan, and oxaliplatin), are generally ineffective and regarded more as palliative therapy for enhancing the life comfort of patients [4,5]. Therefore, there is an urgent need to discover and develop new molecules, drugs, and therapeutic methods to truly help the recovery of PDAC patients after diagnosis in the late stages of disease progression. One of the main problems in PDAC treatment, as mentioned above, is high chemoresistance, which is mostly because of high desmoplasia. Most of the pancreatic cancer mass is rich in collagens, fibronectins, and hyaluronic acid. This mixture of contents creates a very rigid and tough structure that is very hard for single molecules or carriers to penetrate [6,7,8,9]. Additionally, in PDAC, there is a limited amount of blood vessels that can provide a route for therapy, although it is also reported that these blood vessels possess high abnormality in their structure; enhanced permeability and retention (EPR) effects are also observed. This can lead to promising results when a drug-carrier-based strategy is used for potential therapy because one of the principles of nanotechnology in cancer treatment is to use this EPR phenomenon, where a drug carrier leaks out from anomalous cancer blood vessels and passively accumulates in cancer tissues [10,11,12]. Another major challenge in PDAC treatment is the very high metastatic potential, especially in the lungs and liver. It is also confirmed that metastasis occurs very early in cancer progression, where no specific symptoms of the disease are observed [13]. This is also a major problem because PDAC diagnosed at an early stage of progression can be fully treated, as mentioned above. Unfortunately, the vast majority of patients with PDAC are diagnosed at the final stages of the disease, with the predicted average lifespan of 6 months for gemcitabine-treated patients and up to 11 months for those patients who can overcome the strong side effects of FOLFIRINOX therapy [14,15]. These are the reasons why there is a constant need for developing new, effective ways to deal with PDAC and to achieve the full recovery of patients.

One strategy in cancer therapy is to use nanotechnology in the form of drug carriers such as liposomes, nanoemulsions, micelles, or polymeric nanoparticles. Many potentially valuable and promising molecules and compounds are unable to achieve satisfactory plasma concentration values and appropriate concentrations in cancer tissues, which is correlated directly with poor pharmacokinetics and bioavailability parameters [16,17]. By encapsulating drugs in liposomes, for example, doxorubicin, in the form of Doxil, it is possible to elevate drug concentrations in cancer tissues via passive accumulation by the EPR effect [18]. Encapsulation of hydrophobic drugs in more “water-soluble” carriers such as PEGylated liposomes or polymeric nanoparticles can overcome their low bioavailability issue, which is correlated directly with poor water solubility. Apart from liposomes, PLGA nanoparticles are one of the most successfully used nanocarrier systems in the drug delivery and biomaterials industry. Their key asset is their very low toxicity due to hydrolysis in the body to the non-toxic monomers H_2_O and CO_2_ [19]. The possibility of PLGA surface modification by the addition of PEG or specific targeting ligands is also valuable in terms of enhancing therapeutic potential by prolonging blood circulation and active targeting [20]. Different PLGA nanoparticle preparation methods provide many strategies to encapsulate various anticancer drugs, including paclitaxel, doxorubicin, cisplatin, and 5-fluorouracil [21]. With continuing approval of the US Food and Drug Administration (FDA) for PLGA-based nanomedicines, these nanocarriers can be promising alternatives to liposomal drug delivery systems in situations where the encapsulation of certain compounds in liposomes is either inefficient or impossible.

The future of PLGA nanoparticle technology will be the development of hybrid lipid nanoparticles as a novel platform for drug delivery. Bare PLGA nanoparticles are characterized by suitable biocompatibility and biodegradability for pharmaceutical usage, high structural integrity, and stability, with a narrow polydispersity of the particles [22]. However, bare nanoparticles with no proper surface modification will be rapidly cleared from blood vessels by the immune system [23]. The addition of lipids to the PLGA structure results in the production of hybrid lipid/PLGA nanoparticles with a distinct lipid shell and polymeric core. This system creates a bridge between liposomal and polymeric worlds, with benefits coming from both of these worlds. The polymeric core provides excellent structural integrity and stability during storage, biocompatibility, and biodegradability, and the lipid shell provides low immunogenicity using certain lipids, the amphiphilic character of the carrier, and minimal drug leakages during preparation and storage [22]. Using certain preparation methods, there is a possibility of using different lipids to create nanoparticles with the desired lipid shell to obtain certain physiochemical properties of the carrier, such as changing the surface zeta potential from highly negative (for bare PLGA nanoparticles) to highly positive for particles with shells composed of positively charged lipids.

Many medicinal-plant-derived compounds have been tested against pancreatic cell lines. Some of them were reported as highly cytotoxic toward pancreatic cancer (PC) cells, with terpenoids being one of the most interesting groups of compounds. Terpenoids, as a non-water-soluble subclass of natural products, are used in the treatment of skin, lung, colon, and prostate cancer [24], with examples such as docetaxel and paclitaxel used in chemotherapy as apoptosis activators [10,25]. Other reported terpenoids possess various anticancer-specific properties, such as the inhibition of Nf-kB (nuclear factor kappa B) signaling [26,27], the stimulation of proapoptotic caspases 3 and 9 [26], the targeting of DNA damage [24], and apoptosis stimulation [28].

**Figure 1:**
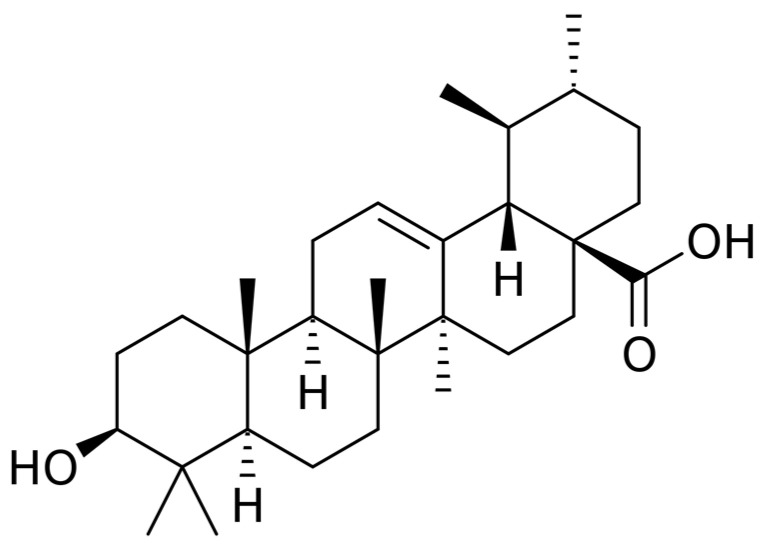
Chemical formula of ursolic acid.

Ursolic acid is a triterpenoid containing six isoprene units; it occurs in a wide variety of medical plants, including rosemary, holy basil, blueberries, cranberries, olives, heather flower, and other higher plants [29,30]. UA possesses a wide range of anticancer properties, e.g., caspase activation [31,32], c-Jun N-terminal kinases (JNK) inhibition [33], downregulation of antiapoptotic genes [34,35], inhibition of COX-2 [36], and suppression of MMP-9 [37]. UA is also reported as a reactive oxygen species (ROS) generator [38], and, more crucially, this terpenoid is reported as an inhibitor of signal transduction and activation of transcription-3 (STAT-3) and Nf-kB, two key cancer-related cell signaling molecules that are closely correlated with PDAC development [39,40,41]. UA can also enhance the therapeutic effect of gemcitabine, which could be beneficial when using UA as a supporting therapy or through a direct combination of UA with gemcitabine as a single chemotherapy [42]. Figure 1 shows chemical structure of UA.

The aim of this study is to design and develop new smart hybrid lipid/PLGA nanoparticles with encapsulated ursolic acid, which we call second-generation nanoparticles, as well as to evaluate their biological activity toward PDAC cell lines. This research is a continuation of our previous work [43], where we prepared the first generation of UA-loaded nanoparticles composed of PLGA with covalently attached manufactured PEG molecules. Our second-generation nanoparticles are lipid-coated PLGA nanoparticles with and without the addition of PEGylated phospholipid. The addition of PEG is crucial for the prevention of rapid reactions between the nanocarrier and the immune system, which results in the clearance of the carrier from blood vessels [44]. We prepared a series of lipid-coated nanoparticles, measured their quality in terms of size and homogeneity, and evaluated weight ratios of lipid to PLGA to achieve the full coating of the carrier. We also measured the long-term stability of these hybrid particles, and we compared them to the first generation of UA-loaded PLGA nanoparticles. Additionally, we established cellular uptake of the hybrid nanoparticles via confocal microscopy and prepared the transmission electron microscopy analysis of selected nanoparticles. Moreover, we performed a hemolysis assay to evaluate their effects on red blood cells. Lastly, we established the cytotoxic potential of the most promising UA-loaded formulation, in terms of size, homogeneity, and stability, toward two PDAC cell lines—metastatic AsPC-1 and primary BxPC-3—and compared their activity with the first generation of our UA-loaded PLGA nanoparticles, providing another basis for further evaluation of this formulation using an in vivo model to gain further insight for potentially effective PDAC treatment in vivo. To the best of our knowledge, this is the first report describing such an approach.

## 2. Results

### 2.1. Ursolic Acid Encapsulation Procedure and Characteristics of Obtained Nanoparticles

Ursolic acid, due to its extremely hydrophobic nature (class IV of the Biopharmaceutics Classification System), is inappropriate in its non-formulated form for intravenous administration. In our previous work [43], we established the first generation of UA-loaded nanoparticles prepared by a nanoprecipitation method, using PLGA as a polymer and 5% Pluronic F-127 as a surfactant, to achieve the appropriate size and polydispersity index (PDI) values of the particles. Additionally, the first generation of the UA-loaded nanoparticles was prepared by slowly pipetting the oil phase into heated and mixed 5% Pluronic F-127. The second generation of UA particles was prepared by slowly injecting the oil phase into the heated and mixed ultrapure water via a syringe. First-generation PEGylated UA-loaded particles were prepared using PEGylated PLGA acquired directly from the manufacturer. The second generation of our UA-loaded particles is composed of PLGA and phospholipids: natural soy phosphatidylcholine and distearoylphosphatidylcholine with PEG 2000, which provides the lipid shell around polymeric nanoparticles. This modification creates hybrid polymeric/lipid nanoparticles that are a bridge between the liposome and polymeric nanoparticle worlds. Using the nanoprecipitation method results in fast and repeatable particle production, with excellent values of size and homogeneity of the sample. The addition of PEGylated phospholipids to the lipid shell composition is necessary to overcome the immunogenic reaction after potential intravenous administration of these hybrid nanoparticles, similar to liposomal STEALTH technology [45]. Moreover, the addition of PEG prevents interaction between particles during the preparation. We have prepared a few different formulations of ursolic-acid-loaded and -unloaded particles with different lipid shell compositions (different in DSPE-PEG 2000 contribution to the lipid shell). Table 1 presents the UA-loaded nanoparticle compositions and their abbreviations used in our experiments.

**Figure 2:**
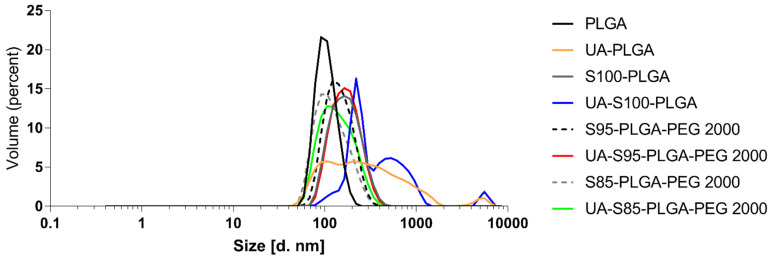
Size distribution of the tested nanoparticles.

**Figure 3:**
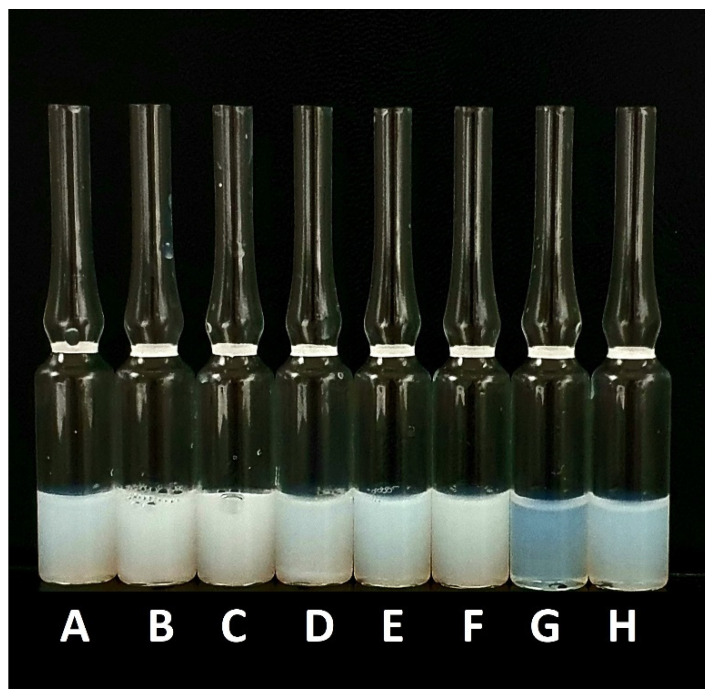
Visual appearance of the obtained nanoparticles: (**A**) PLGA, (**B**) UA-PLGA, (**C**) S100-PLGA, (**D**) UA-S100-PLGA, (**E**) S95-PLGA-PEG 2000, (**F**) UA-S95-PLGA-PEG 2000, (**G**) S85-PLGA-PEG 2000, and (**H**) UA-S85-PLGA-PEG 2000.

After the formation of the particles, the samples were cooled down and separated from DMSO residues and non-encapsulated UA by gel filtration using Sephadex G-50 microcolumns. For this experiment, for all formulations, UA was loaded using a compound-to-PLGA weight ratio of 1:10. As shown in Table 2, the dynamic light scattering results indicated that the diameter of the UA-loaded nanocarriers ranged from 145.1 ± 2.6 nm for UA-S85-PLGA-PEG 2000 hybrid nanoparticles to 206.8 ± 3 nm for non-PEGylated, bare UA-PLGA nanoparticles. Additionally, PDI values ranged from 0.08 ± 0.02 for UA-S95-PLGA-PEG 2000 hybrid nanoparticles to 0.63 ± 0.1 for the UA-S100-PLGA sample. The zeta potential values ranged from 30.4 ± 0.3 for UA-PLGA nanoparticles to −42 ± 1.2 for UA-S85-PLGA-PEG 2000 hybrid nanoparticles. Unloaded nanoparticles were also prepared and measured. There is a visible difference between unloaded and loaded particles, where packing UA into particles results in an increase in the size of the particles. (an additional 20.9 nm for the S95 formulation and 14.8 for the S85 formulation). Figure 2 presents the size of the distribution for each prepared sample. There is a notable difference between loaded and loaded hybrid particles, with increased size for loaded particles. UA loading into S100-PLGA particles resulted in the rapid aggregation of the sample, and this formulation was not used in the next experiments. Additionally, the zeta potential of this sample was not measured due to unacceptable size and PDI values. Figure 3 presents a photograph of each PLGA nanoparticle sample obtained and measured in this experiment.

### 2.2. Stability Studies of UA-Loaded and Unloaded Nanoparticles

**Figure 4:**
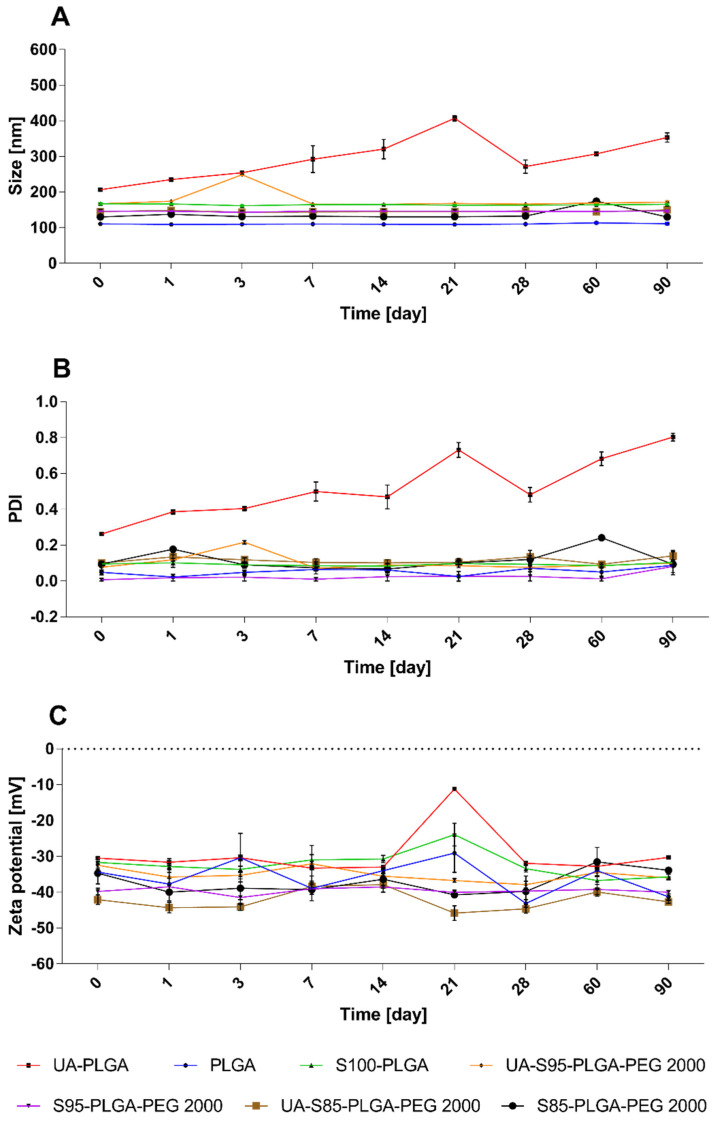
Stability of different bare and coated, non-loaded and loaded nanoparticles: changes in size graph (**A**), PDI graph (**B**), and zeta potential graph (**C**) values.

The long-term stability of nanocarriers is a very important parameter. Every nanoformulation must maintain its morphology values, and samples must not aggregate or lose their payload. We performed the long-term storage of samples under 4 °C and measured their size, PDI, and zeta potential values at each fixed time point. The results of these measurements are presented in Figure 4. Most of the measured samples retained their initial values of size, PDI, and zeta potential for the whole period of the experiment, with minor changes. Bare UA-PLGA nanoparticles prepared by this method lost their homogeneity and proper size over the time of the experiment, probably due to the aggregation of the particles or the precipitation of ursolic acid from the carrier. Their size increased from 206.8 ± 3 to 353.4 ± 12 nm. The PDI parameter for this sample was also unstable and rose from 0.26 ± 1 to 0.8 ± 0.02. For lipid-coated samples, S100-PLGA retained its size and homogeneity during the experiment, but the UA-S100-PLGA sample was unobtainable due to its aggregation during the preparation of the particles, and its stability was not evaluated. Every coated and PEGylated, loaded and unloaded hybrid carrier was stable during the 90 days of the experiment. Because of the best values of the sample and PDI, for further experiments, we chose the UA-S85-PLGA-PEG 2000 sample. This sample was characterized by excellent stability, proper size, and homogeneity and was suitable for intravenous administration.

### 2.3. Hybrid UA-Loaded Nanoparticle Serum Stability Assay

**Figure 5:**
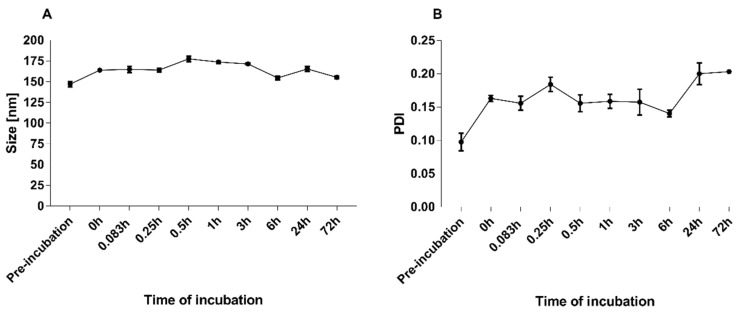
Stability of UA-S85-PLGA-PEG 2000 hybrid nanoparticles over 72 h of incubation with 50% solution of FBS: (**A**) change in the size of the nanoparticles, (**B**) change in PDI values of the nanoparticles.

**Figure 6:**
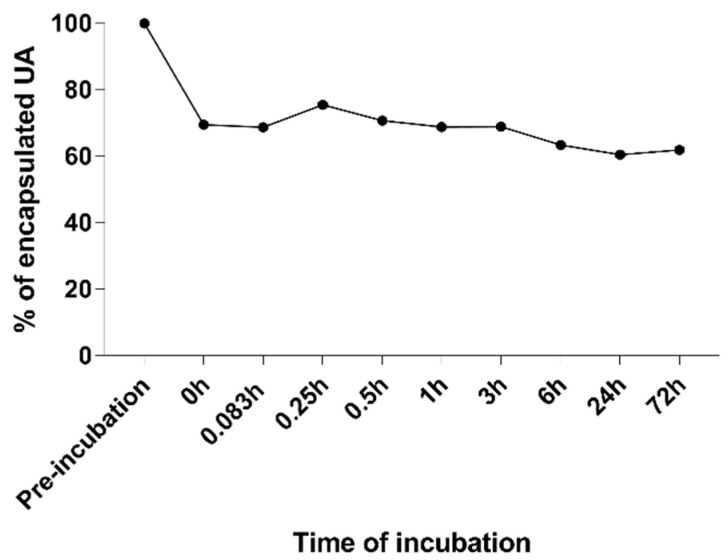
Evaluation of payload loss over 72 h incubation of UA-S85-PLGA-PEG 2000 nanoparticles with 50% FBS.

For drug delivery systems, it is necessary to establish their morphological parameters and retention ratios during incubation with serum proteins. For this experiment, the UA-S85-PLGA-PEG 2000 sample was incubated for 72 h with 50% FBS solution at 37 °C. At fixed timepoints, 200 uL of nanoparticles/FBS suspension was gathered and separated using sepharose-4B minicolumns equilibrated with MILIQ ultrapure water. After purification of the sample from serum proteins, we established size and PDI values as well as the concentrations of UA left inside the nanoparticles. The results are presented in Figure 5 and Figure 6. UA-loaded nanoparticles retained almost 70% of their payload during the 72 h of incubation with serum proteins. Initial loss at the beginning of the experiments suggests a rapid leakage of the UA fraction encapsulated between acyl chains of the lipid coat. During the 72 h of the experiment, the particles did not change their size and homogeneity parameters, which suggests overall satisfactory bloodstream stability of the sample during potential intravenous administration.

### 2.4. Hybrid UA-Loaded Nanoparticle Characterization: Coating Dynamics and Encapsulation Efficiency Evaluation

**Figure 7:**
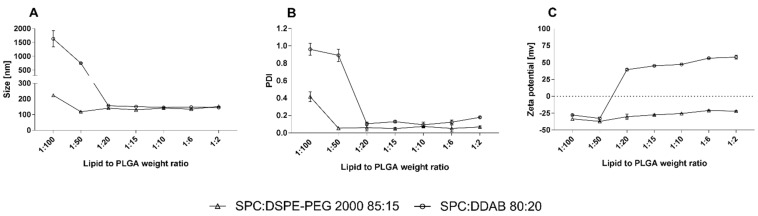
PLGA nanoparticle-coating dynamics with phospholipids. Graph (**A**): size value changes with the increasing amount of phospholipids used. Graph (**B**): PDI value changes with the increasing amount of phospholipids used. Graph (**C**): zeta potential value changes with the increasing amount of phospholipids used.

In our study, it was necessary to establish the ratio between PLGA and phospholipids to achieve the full coating of PLGA particles. This value was achieved by measuring changes in size, PDI, and zeta values. The plateau of these values was treated as a full coating. For this assay, two model types of lipid coat compositions were used: SPC:DSPE-PEG 2000 (85:15 molar ratio) and SPC:DDAB (80:20 molar ratio). The results of these experiments are presented in Figure 7. For both compositions, a full coating is observed for the lipid-to-PLGA weight ratio of 1:6, where a plateau of zeta potential and size is achieved. Therefore, this ratio was used in further hybrid nanoparticle preparation.

**Figure 8:**
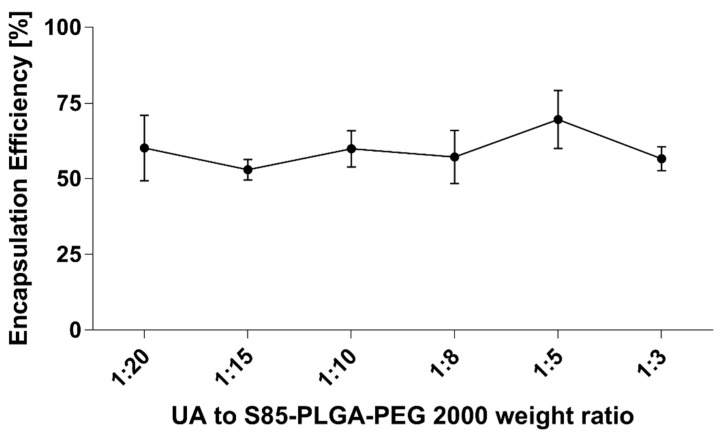
Ursolic acid encapsulation efficiency with different UA-to-S85-PLGA-PEG 2000 weight ratios.

We also established the encapsulation efficiency of UA loaded into PLGA hybrid nanoparticles with a SPC:DSPE-PEG 2000 85:15 phospholipid coat. During our experiments, this formulation, named UA-S85-PLGA-PEG 2000, was characterized by the best stability parameters, and it was chosen for the next cellular assays. We encapsulated UA using a fixed weight ratio of UA to polymer, ranging from 1:20 to 1:3. The encapsulation efficiency results are presented in Figure 8. The results show that the way of UA encapsulation was independent of the UA-to-polymer weight ratio. The encapsulation efficiency ranges from 53% ± 3.4 to 69.6% ± 9.5, with no correlation with the weight ratio used for encapsulation. This result suggests the spontaneous nature of UA encapsulation into hybrid nanoparticles, where UA molecules are entrapped into spheres during their formation after injection.

**Figure 9:**
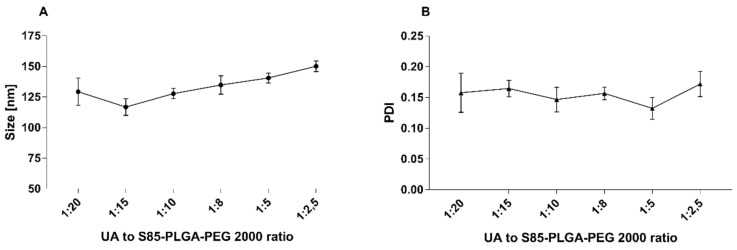
Changes in the size graph (**A**) and PDI graph (**B**) of UA-S85-PLGA-PEG 2000 hybrid nanoparticles with an increasing amount of UA used in sample preparation.

We also measured the size and PDI changes of the loaded particles during UA loading. The results presented in Figure 9 clearly show the increasing size of the particles with an increasing amount of UA loading into the particles.

### 2.5. Transmission Electron Microscopy (TEM) Visualization of Nanoparticles

**Figure 10:**
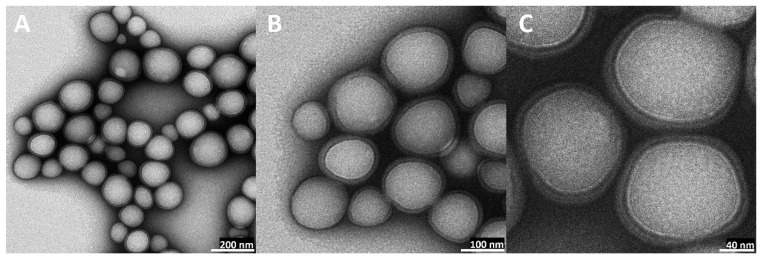
TEM images of bare PLGA nanoparticles: (**A**): magnitude 50 k, scale bar 200 nm; (**B**): magnitude 100 k, scale bar 100 nm; (**C**): magnitude 200 k, scale bar 40 nm.

**Figure 11:**
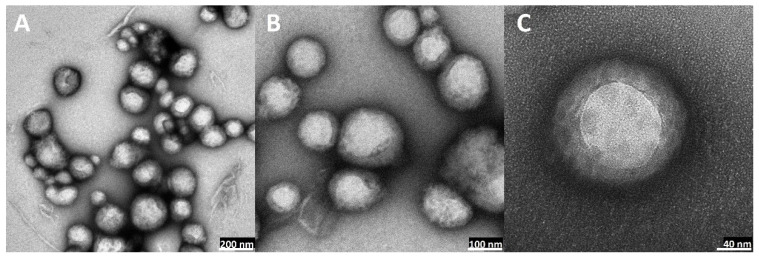
TEM images of bare UA-loaded PLGA nanoparticles: (**A**): magnitude 40 k, scale bar 200 nm; (**B**): magnitude 80 k, scale bar 100 nm; (**C**): magnitude 200 k, scale bar 40 nm.

**Figure 12:**
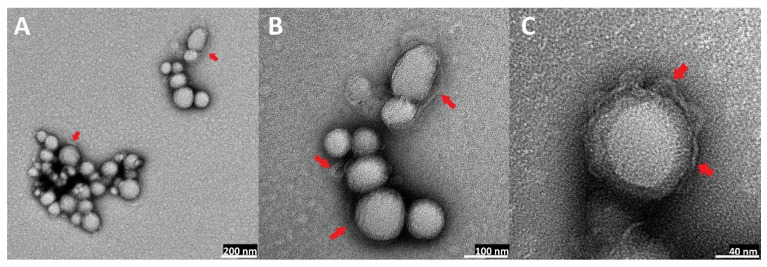
TEM images of non-loaded hybrid S85-PLGA-PEG 2000 nanoparticles: (**A**): magnitude 40 k, scale bar 200 nm; (**B**): magnitude 100 k, scale bar 100 nm; (**C**): magnitude 250 k, scale bar 40 nm. Red arrows indicate lipid layers coating PLGA particles.

**Figure 13:**
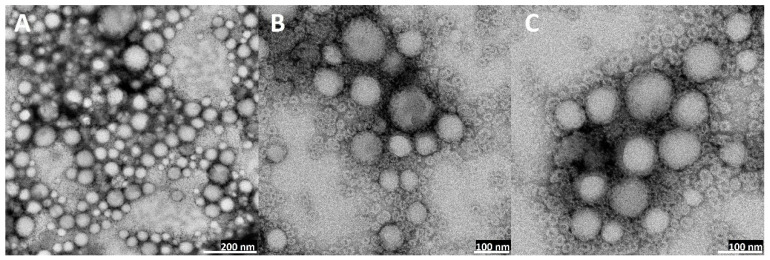
TEM images of UA-loaded hybrid S85-PLGA-PEG 2000 nanoparticles: (**A**): magnitude 30 k, scale bar 200 nm; (**B**): magnitude 80 k, scale bar 100 nm; (**C**): magnitude 120 k, scale bar 100 nm. Red arrows indicate lipid layers coating PLGA particles. Around the particles, there is a clearly visible layer of small phospholipid vesicles.

The visual appearance of the UA nanoparticles in the solution was translucent, similar to very diluted milk, but still very transparent (Figure 3). For microscopic visualization, TEM was used. It was very important to visually evaluate the difference between plain PLGA UA-loaded and unloaded particles and compare them to the coated, hybrid nanoparticles. TEM images (Figure 10, Figure 11, Figure 12 and Figure 13) show spherical entities with good homogeneity. However, the most important information is the visual appearance of the lipid coat. In unloaded S85-PLGA-PEG 2000 particles, the lipid coat is an integral part of the particles but retains some fluidity, similar to liposomes composed of soy lecithin. In the UA-S85-PLGA-PEG 2000 sample, this lipid coat forms structures similar to very small liposomes; some of them are an integral part of the nanostructure, while others tend to disconnect from the particles.

### 2.6. Cellular Uptake of UA-S85-PLGA-PEG 2000 Nanoparticles

**Figure 14:**
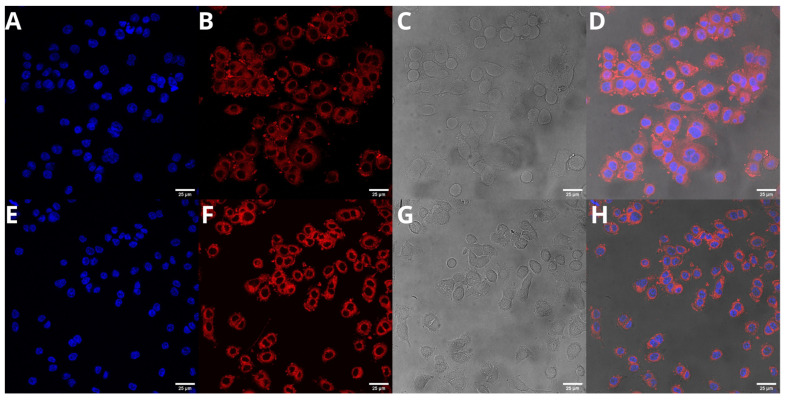
Visualization of the cellular uptake of Nile-Red-loaded UA-S85-PLGA-PEG 2000 nanoparticles by AsPC-1 pancreatic cell lines (**A**,**E**). DAPI (**B**,**F**). Nile Red fluorescence signal (**C**,**G**). Transmitted light (**D**,**H**). The merged image of the cells was observed after 15 (**A**–**D**) and 120 min (**E**–**H**) of incubation with the labeled particles. Scale bar = 25 µm.

**Figure 15:**
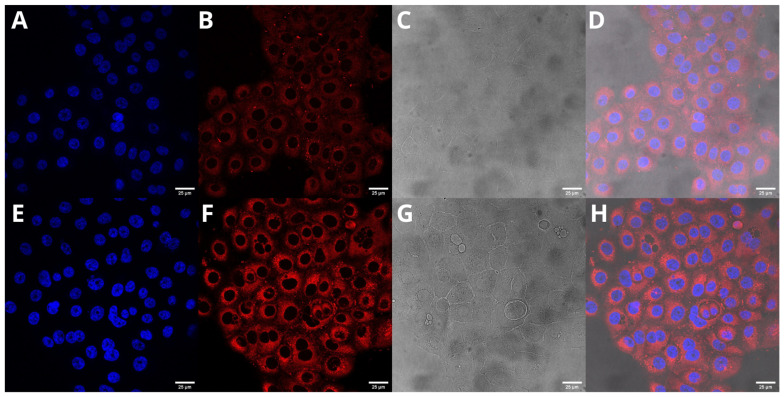
Visualization of the cellular uptake of Nile-Red-loaded UA-S85-PLGA-PEG2000 nanoparticles by BxPC-3 pancreatic cell lines (**A**,**E**). DAPI (**B**,**F**). Nile Red fluorescence signal (**C**,**G**). Transmitted light (**D**,**H**). The merged image of the cells was observed after 15 (**A**–**D**) and 120 min (**E**–**H**) of incubation with the labeled particles. Scale bar = 25 µm.

**Figure 16:**
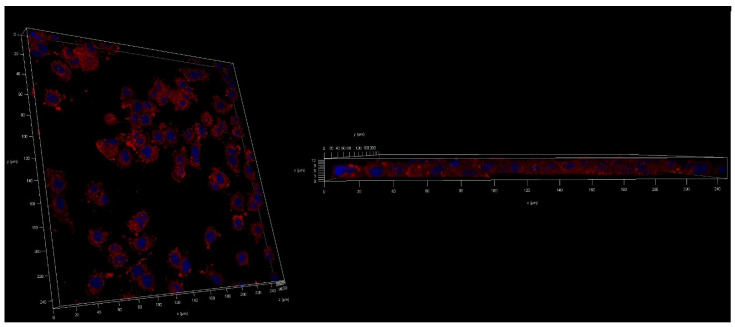
Z-stack analysis of the cellular uptake of Nile-Red-loaded UA-S85-PLGA-PEG 2000 nanoparticles by AsPC-1 pancreatic cell lines.

**Figure 17:**
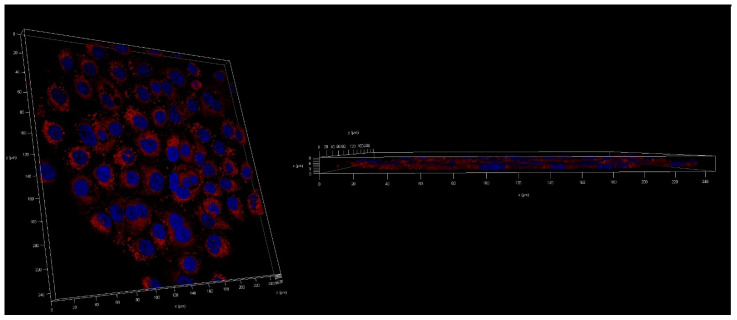
Z-stack analysis of the cellular uptake of Nile-Red-loaded UA-S85-PLGA-PEG 2000 nanoparticles by BxPC-3 pancreatic cell lines.

The next step was to evaluate the cellular uptake of the nanoparticles. For this purpose, we labeled nanoparticles with Nile Red, which is commonly used for bioimaging studies [46]. Confocal microscopy observation was performed using fluorescence signals from two fluorophores: one from cell nuclei stained with DAPI and the second from Nile Red encapsulated in nanoparticles, with the addition of transmitted light. We performed this assay for 15 and 120 min. The results, presented in Figure 14 and Figure 15, show that the particles were effectively internalized within the AsPC-1 and BxPC-3 cells after this incubation period. These results were also confirmed by Z-stack internalization analysis, where fluorescence signals from the labeled particles were gathered across the cellular space. These results are presented in Figure 16 and Figure 17.

### 2.7. Assessment of UA-S85-PLGA-PEG 2000 Nanoparticles’ Toxicity toward Human Pancreatic Cancer Cell Lines

**Figure 18:**
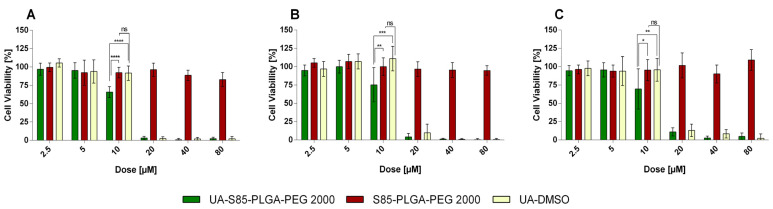
Cytotoxic effect of ursolic acid loaded into S85-PLGA-PEG 2000 hybrid nanoparticles or in the free form in DMSO, as determined by MTT assay, after 72 h of incubation for AsPC-1 panel (**A**), BxPC-3 panel (**B**) and NHDF panel (**C**) cell lines. Non-loaded hybrid particles were tested as well. For a dose of 10 μM, the statistical significance of differences between free and loaded compounds evaluated by GraphPad Prism 7 is indicated by asterisks (0.1234 Ns, 0.0332 *, 0.0021 **, 0.0002 ***, >0.0001 ****), with 95% confidence. Ns stands for “non-significant”.

To evaluate the anticancer potential of the UA and UA-S85-PLGA-PEG 2000 nanoparticles, we investigated their in vitro cytotoxicity against two human pancreatic cancer cell lines (AsPC-1 and BxPC-3). The nanoparticles were also tested on a normal, dermal, human fibroblast cell line (NHDF). During the experiments, cells were incubated for 72 h with UA in DMSO solution (free compound), UA loaded into hybrid nanoparticles, and unloaded particles (without UA). The experimental outcome was established using the MTT test, which is based on the detection of the oxidoreductive enzymes (especially succinate dehydrogenase) in the mitochondria of living, fully metabolizing cells. During the experiment, cells were incubated with a range of concentrations (2.5–80 µM) of UA dissolved in DMSO (which is commonly used as a solvent for drug testing), which was treated as a positive control, or UA encapsulated in S85-PLGA-PEG 2000 hybrid nanoparticles. As negative controls, non-loaded nanoparticles were used. DMSO, as another negative control, was tested in our previous experiment, where it was established as non-toxic in the same experiment conditions [43]. The results are presented in Figure 18, whereas the calculated IC50 values are presented in Table 3.

### 2.8. Hemolysis Assay

To detect the possible hemolytic activity of UA-S85-PLGA-PEG 2000, a hemolysis assay was performed. This test was based on the estimation of the level of released hemoglobin from erythrocytes after incubation with nanoparticles. It was found that after contact with UA-S85-PLGA-PEG 2000 at a concentration corresponding to the IC50 (12 µM) of the nanoparticles, the lysis of human red blood cells was very low (2.9 ± 0.4%). This negligible degree of hemolysis is within an acceptable range to be considered nonhemolytic material.

## 3. Discussion

Pancreatic ductal adenocarcinoma is the most commonly diagnosed type of pancreatic cancer, and despite all the efforts, research, and therapies, it is still known as one of the most deadly types of cancer. There is prolonged, non-specific progression, being diagnosed mostly in the final stages, with a predicted average lifespan after diagnosis of up to 6 months and a small fraction of patients being suitable for more advanced therapies, which will give additional months of lifespan [47]. Diagnosis of PDAC is commonly treated as a death sentence. With the present medicine, there is no available effective therapy to fully cure PDAC in the late stages of disease progression. The only known way to successfully combat PDAC is to test our bodies systematically and prophylactically, maintain a healthy diet, and lead a healthy lifestyle due to the direct correlation between PDAC morbidity and lifestyle [1]. 

That is why there is a need to focus on future medicine and possibilities: discovering and delivering new molecules, drugs, nanocarriers, and whole therapy systems to fully cure patients in the late stages of PDAC, where most diagnoses occur. One of the strategies to deal with such an aggressive disease is to use naturally derived compounds that possess a wide spectrum of anticancer activities that help to overcome multidrug resistance [48,49], inhibit metastatic events [50], inhibit precancerous signaling pathways [51,52], or exert a direct cytotoxic effect on cancer cells [53,54]. However, the biggest obstacle to the direct usage of these naturally derived molecules is their poor bioavailability due to their hydrophobic nature, which, in consequence, disqualifies them from direct intravenous usage without a specific nanoformulation [55].

Ursolic acid is a member of a large subclass of naturally derived terpenoids, which includes some well-known anticancer drugs such as paclitaxel and docetaxel. UA possesses various interesting and promising anticancer properties, such as direct cytotoxic, antimetastatic, and chemopreventive effects [34,38,39]. It is very interesting that there is a possibility for chemotherapy where UA is combined with gemcitabine, the most popular and common chemotherapeutic used in PDAC treatment [56]. UA is reported to enhance the bioactivity of gemcitabine towards cancer cells and to overcome the very poor response from gemcitabine therapy alone [57]. Additionally, with the successful liposomal encapsulation of gemcitabine reported [58] and a confirmed synergistic effect between these two compounds [42], there is a possibility of establishing a chemotherapy protocol using a combination of gemcitabine and UA, based on nanotechnology, where two types of PEGylated nanocarriers can accumulate in cancer-associated tissues via the EPR effect.

PLGA-based nanoparticles are emerging as promising alternatives to liposomes as pharmacological drug carriers. They possess characteristics suitable for intravenous administration, such as appropriate size, biocompatibility, and the possibility for surface modification, to enhance their potential anticancer properties. They are also non-toxic due to their metabolism of water and carbon dioxide [19,59,60]. Additionally, similar to liposomes, there is a possibility to prepare PLGA nanoparticles with PEG modification: either directly to PLGA chains in a block–block manner or via the addition of PEGylated phospholipids to the systems, which results in the creation of a lipid layer around PLGA particles. The possibility of delivering a therapeutic agent into PLGA particles via oral, pulmonary, or intravenous routes creates an interesting system for developing various nanocarrier-based drugs.

This work is a continuation of our previous approach to developing an effective bioactive nanocarrier for PDAC cells. As mentioned above, the first attempts to encapsulate UA into liposomes resulted in the achievement of high entrapment efficiency but without significant biological activity from the formulation. This phenomenon is still not explained [43]. That is why we have focused on a different approach with UA encapsulation. We chose PLGA polymers to prepare the ”first generation of UA-PLGA nanoparticles”, as we call them in the context of this work. Previously, we prepared UA-PLGA nanoparticles using a nanoprecipitation method with the addition of Pluronic F-127 as a surfactant to prevent non-specific interaction between particles, especially in the case of the non-PEGylated variant. We established a protocol for the preparation of three different types of particles: one sample with bare PLGA nanoparticles with encapsulated UA, and two samples with the polyethylene glycol addition with two different chain lengths: 2000 and 5000 Da. This resulted in the successful preparation of three different bioactive nanocarriers based on PLGA with encapsulated UA. These samples were characterized by good size and homogeneity values, but after 33 days of long-term stability assay, there was a visible change in the size of the particles, probably due to the direct effect of water on the particles [61,62].

As an evolution of the first generation of UA-PLGA particles, we decided to produce the “second generation of UA-PLGA nanoparticles”. These particles are composed of PLGA polymers and phospholipid monomers. This combination results in the creation of novel hybrid nanoparticles, where PLGA polymers with encapsulated UA take part as the core of the particles and phospholipids create a lipid shell around the polymeric core. This innovative approach results in the creation of a lipid–polymeric hybrid, a bridge between liposomal and polymeric nanoparticle worlds. Coating PLGA nanoparticles with various phospholipids creates an opportunity to modify the morphological parameters of the particles, for example, by coating negatively charged PLGA nanoparticles with a mixture of lipids composed of soy lecithin and positively charged cationic phospholipids such as DOTAP or DDAB, as in our work. This leads to a change of the zeta potential value of the particles from negative to positive. This aspect may suggest the use of positively charged hybrid PLGA/lipid particles in potential gene therapies, similar to liposomes composed of DOTAP [63]. In the context of this work, the most important aspect is the addition to the lipid coat of DSPE-PEG 2000, a phospholipid commonly used in the preparation of liposomal formulations of the widely known cytostatic agent doxorubicin (Doxil). Coating the PLGA nanoparticles with a lipid shell, whose composition resembles those used in the drug Doxil, results in the translation of liposomal stealth technology to the PLGA nanoparticle environment, with benefits including the reduction of unwanted interaction between coated particles (similar to non-PEGylated liposomes composed of pure soy lecithin) and, most importantly, PEGylation of the particles, significantly reducing interaction with the reticular–endothelial system and macrophages, which results in better pharmacokinetics of the carrier due to the leveling of rapid blood clearance by elements of the immune system [64,65].

During the preparation of hybrid nanoparticles, once again, we chose the nanoprecipitation method, with polymer and phospholipids of a certain composition dissolved in DMSO as the oil phase and ultrapure water as the water phase. This one-step method is characterized by good reproducibility and scalability with controllable preparation conditions but relatively low encapsulation efficiency [66]. However, this method can provide high-quality samples of nanoparticles with a narrow polydispersity index and appropriate size for intravenous administration [67]. Our updated procedure was successful in obtaining a series of bare and lipid-coated nanoparticles with and without encapsulated ursolic acid. Every PEGylated (5 or 15 mol%) formulation represents values of size and homogeneity, ranging from 130.4 to 167.3 nm, with PDI values below 0.1. These parameters, together with benefits derived from the PEGylated phospholipid coating, led to obtaining an excellent candidate for a potential new anti-pancreatic cancer intravenous drug. Compared with our previous work, second-generation UA particles are represented by similar particle sizes to the first generation (where size values ranged from 132 to 168), although PDI values for the second generation are not higher than 0.1 compared to the first generation (where PDI values were not higher than 0.2). We also prepared particles with a coating made from pure soy lecithin. Unloaded particles are represented by good values of size (167.2 nm) and PDI (0.1), but, unfortunately, loading UA into this type of particle results in the massive aggregation of the sample, probably due to undesirable interaction between the compound, phospholipid, and polymer during particle formation. Bare particles were also prepared, but with ultrapure water as the water phase instead of 5% Pluronic F-127. These particles are characterized by higher values of size (206.8 nm) and PDI (0.26) for UA-loaded PLGA particles, whereas our first variant was characterized by more suitable parameters (size 167.1 nm, with PDI 0.128). Additionally, their stability during long-term storage is much worse than the first generation. For the second generation, after 90 days of storage, we observed an increase in size (from 206.8 to 353.4 nm) and PDI value (from 0.26 to 0.8), which is correlated with either the aggregation of the sample due to the lack of the surface protective properties of Pluronic F-127 or, more likely, the precipitation of the compound from the particles. Because there was no significant difference in bare, non-loaded PLGA particles prepared in ultrapure water in terms of size and PDI and also the stability of PEGylated, lipid-coated nanoparticles, we believe that it is necessary to use surface protection to prevent aggregation and undesirable compound release from the particles. This surface protection can be used in the form of surfactants such as Pluronic F-127, F-68, or a phospholipid coat.

Additionally, with the second generation of UA-loaded particles, the use of phospholipids enhanced the encapsulation efficiency values of the compound. With the first generation, we performed loading with a fixed UA-to-PLGA weight ratio of 1:10, with efficiencies ranging from 43.1% to 47.4%. In the context of this work, we further explored the dynamics of UA encapsulation into nanoparticles with various UA-to-PLGA ratios. The addition of a lipid coat in the second generation of UA-loaded particles resulted in increased values of encapsulation efficiency, from 53% to 69.6%. This increase could be correlated with the entrapment of some of the fraction of the UA between the acyl chains of the lipid layer coating the particles; a similar phenomenon is known from the encapsulation of compounds in a liposomal bilayer [68,69]. A very important aspect of this assay is the phenomenon where, no matter what UA-to-polymer weight ratio is used, the value of the encapsulation efficiency is the same; the diagram is somewhat flat in this matter. With the correlation of size measurements, where an increasing size of the compound applied for nanoformulation is used, the size of the particles increases. During this loading, we can assume that with this method, the loading process is rather spontaneous: the compound is encapsulated directly during the formation of the particles and the loading efficiency is fixed within some specific value range. We believe that this phenomenon is linked to the technology used and the method itself (nanoprecipitation), where there are limited possibilities for condition optimization. Perhaps methods using microfluidic systems could be helpful in increasing encapsulation efficiency values [70,71].

The long-term stability of the samples is a very important aspect of describing their quality and potential for future application. First-generation nanoparticles were characterized by good long-term stability, but after 33 days of incubation, there was a clearly visible increase in size (of about 15 nm more per measured sample) due to the negative impact of water on the PLGA particles [66]. We did not observe a major change in the homogeneity of the sample; more likely, there was a “swelling” and expansion of each particle without losing the homogeneity of the whole population of particles. With the second generation of our nanoparticles, we did not observe any major disturbance in terms of size, homogeneity, and zeta potential values of the samples even after 90 days, with the exception of UA-loaded bare nanoparticles, as mentioned above. These results correlate with previous data, where the lipid coat prevented interaction between the water environment and the polymer core due to the hydrophobic character of the coat [22].

Another important aspect of drug delivery systems evaluation is their stability in the presence of plasma proteins. It is necessary to develop formulations where bonding to serum proteins is strictly limited or fully prevented. Too strong an interaction between proteins and nanoparticles could lead to the rapid clearance of the particles from the bloodstream, with poor pharmacokinetics as a result [72]. The introduction of PEG was a major breakthrough in terms of enhancing bioavailability, increasing the pharmacokinetic and therapeutical potential manyfold by preventing interactions between the PEG-ylated liposomes and serum proteins [18]. In our studies, we have incubated our UA-loaded hybrid nanoparticles with 50% FBS solution for 72 h hours. The initial loss of payload, immediately after the injection of the hybrid particle suspension into the heated solution of FBS, suggests that some of the UA could be encapsulated between the acyl chains of the phospholipid coat, similar to passive-loading drug encapsulation in liposomes [68,69]. This could also suggest increased values of UA encapsulation efficiency into hybrid lipid–polymeric nanoparticles compared to bare particles from our previous work [43]. There is also a significant change in the size of the particles immediately after injection into FBS solution; however, the size parameter, as well as the homogeneity value described by the PDI value, did not change significantly during the time of the experiment; it slightly increased its value up to 0.2 but maintained its adequate parameters for intravenous dosage [73].

**Figure 19:**
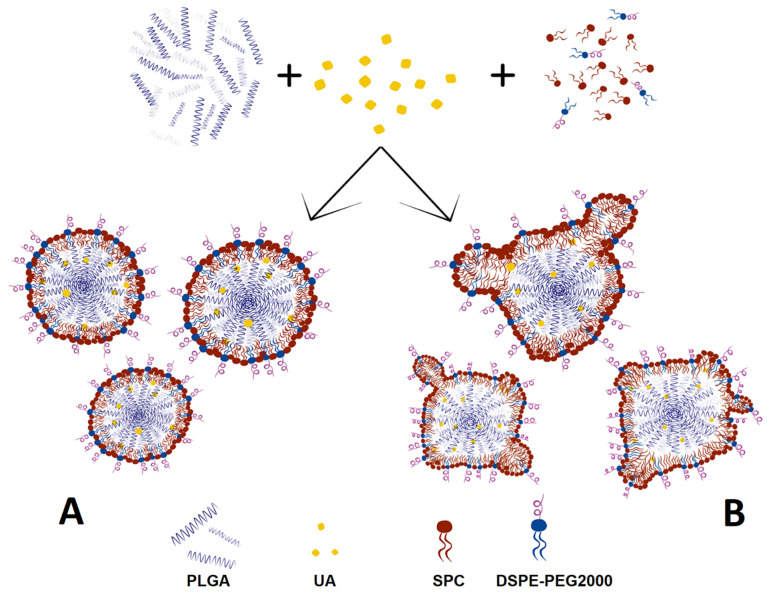
Comparison between the theoretical, “ideal” structure of UA-S85-PLGA-PEG 2000 nanoparticles (**A**) and that observed by TEM (**B**).

In the context of forming hybrid lipid/polymer nanoparticles, it is very important to confirm that the coating of the particles is successful. Some sources [22,74] have proposed that we should suspect very organized lipid structures around particles, in the form of mono- or bilayers, depending on the method used for preparation. Other sources [75,76] have proposed that phospholipids will create very nonhomogeneous but still integral structures in the form of bubbles or “butterfly wings”. In our work, we can confirm the second thesis, where we observed, analyzing TEM photographs, that the phospholipid maintains some of the characteristics of the SPC: its fluidity at room temperature, similar to liposomes made from this phospholipid. Additionally, the addition of PEGylated phospholipids in the form of distearoylphosphatidylethanoloamine with conjugated 2000 Da PEG results in the formation of “microbubbles”, very similar to liposomes but much smaller. These structures, with a size of around 10 nm, are an integral part of nanoparticles, with some of them tending to back off from the particles into the water solution. This phenomenon, in our opinion, needs to be explained and subjected to more extensive analysis, especially to establish the definitive structure of lipid coatings by additional advanced electron microscopy. Figure 19 shows a comparison between the theoretical and ideal shape of a phospholipid coat around particles and the actual shape based on TEM microscopy analysis.

**Figure 20:**
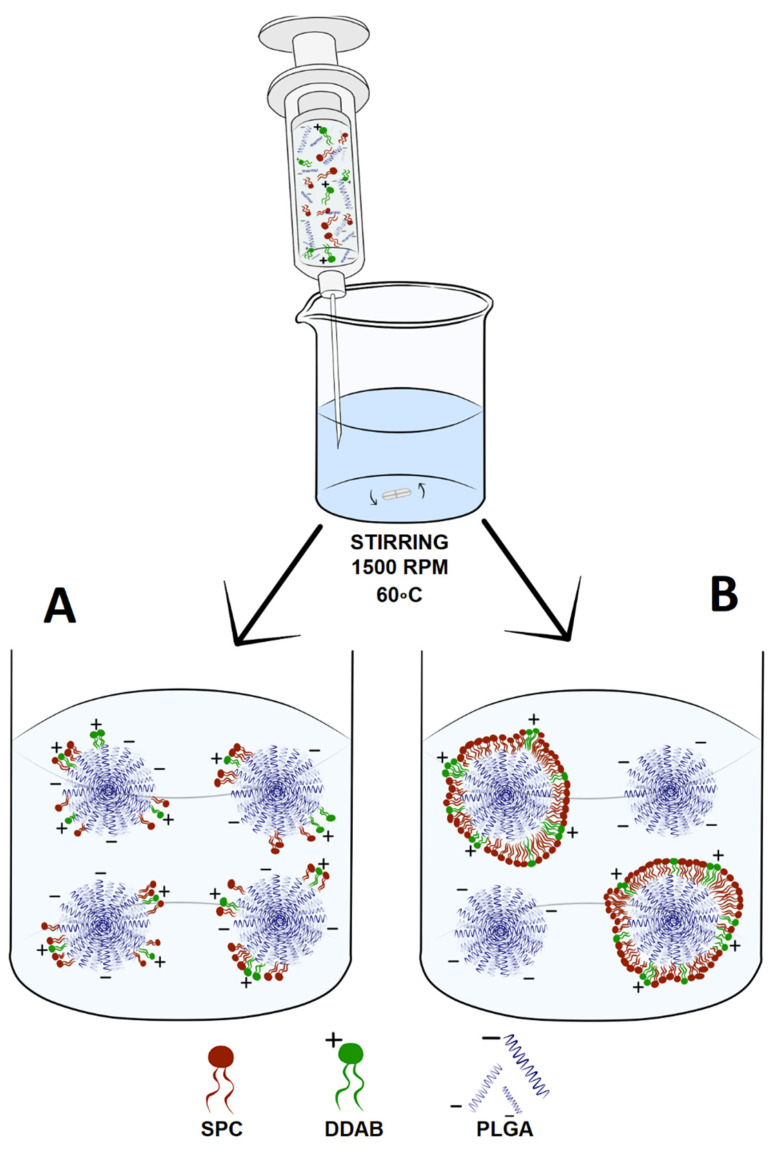
Proposed mechanism of PLGA coating with positively charged lipid layers according to zeta potential measurements for the 0 mV point, where (**A**) shows a variant with the coating of every PLGA particle equally with fractions of cationic lipids to full coating at the point of saturation, where a zeta potential plateau is achieved, and (**B**) shows a variant with the sequential full coating of every single PLGA particle with an increasing amount of lipids used.

Another crucial aspect is to establish a point in the lipid-to-PLGA weight ratio where we can achieve a full coating of plain PLGA nanoparticles. For this assay, we used two different lipid compositions: SPC:DSPE-PEG 2000 with an 85:15 molar ratio and SPC:DDAB with an 80:20 molar ratio. The first one is the “default” lipid composition, treated as being the best in terms of preparation, stability, morphological parameters, and biologically active UA-loaded nanoparticles. The second composition consists of positively charged, cationic lipid DDAB. The idea of using positively charged lipids in this experiment was to achieve an additional, more radical change in zeta potential value, from highly negative (−40 mV) to highly positive (+45 mV), after the full coating of the PLGA particles. During this experiment, we established a lipid-to-PLGA weight ratio of 1 to 6, similar to Zhang et al. [77]. However, with the cationic lipid coat, there was a complete charge shift from highly negative to highly positive. This confirms that by using a phospholipid, we can manipulate the potential of the carrier. These positively charged hybrid nanoparticles could be used as gene transporters, similar to cationic liposomes [63]. Further important information gained from this assay is the overall process of the coating. We suspected that lipids would be organized in a “zero–one” manner or “all or nothing”, similar to liposome or micelle formation from lipophilic monomers, after achieving the critical micellization concentration (CMC) [78]. In this case, especially with the coating using DDAB in its composition, at the point where the zeta potential value equals zero, we suspect that half of the particles will be coated and the other half not, and the overall charge will be leveled. Figure 20 shows the two proposed variants of particle-coating dynamics. We believe that this aspect of hybrid lipid nanoparticles should be investigated in the near future.

Additionally, we performed a cytotoxicity assay to establish the overall anticancer potential of our formulation. We chose the UA-S85-PLGA-PEG 2000 formulation for this experiment due to it having the best morphological and stability results. This formulation is characterized by IC50 values of 8.6 µM for the metastatic AsPC-1 cell line and 12.7 µM for primary BxPC-3 cell lines. Moreover, these values are significantly lower than the IC50 values of UA dissolved in DMSO as a positive control. According to our first generation of UA-PLGA nanoparticles, the IC50 values are similar between the two generations of particles [43]. Additionally, there is no toxic effect coming from the unloaded carrier, which is very important in terms of evaluating any drug-carrier-based biological activity. We also evaluated the cytotoxic potential of our hybrid particles on a normal NHDF cell line. Based on IC_50_ values, we did not observe any significant specificity of our UA-loaded hybrid particles towards pancreatic cell lines. However, due to the nature of the sample, this result does not disqualify this nanocarrier as potentially valuable ani-PDAC medicine because of the different fates of the drug carrier between intravenous administration and direct administration to the 2D cell culture on the microplate. Nanocarriers will accumulate in cancer-associated regions, according to the EPR effect, and minimize adverse and side effects from the bioactive compound itself [79,80]; 2D assays, such as the MTT assay, are the very first step in developing new drug candidates, and our goal is to evaluate the cytotoxicity of these candidates towards specific cancer-related cell lines. There are major differences in cell response between 2D cell cultures and 3D models in the form of spheroids, organoids, and living organism models [81,82]. 

Cellular uptake assays using confocal microscopy confirm that our second-generation UA-loaded particles are successfully internalized in cellular space and release their payload directly into cells instead of being digested in the cell medium. We evaluated two different timepoints for this experiment: 15 and 120 min. Our results match the data obtained by other groups. Cartiera et al. [83] confirmed the successful internalization of PLGA nanoparticles with encapsulated rhodamine 6G (Rhod6G) into the cellular space of OK cells from 1 to 24 h of incubation with Rhod6G-loaded nanoparticles, with a slight rise in fluorescence signal. Xu et al. [84] performed another experiment using OVCAR-5 cells, where the cells were incubated with nanoparticles with encapsulated Nile Red. They reported the successful internalization of the samples even after 1 min of incubation, with a significantly higher signal after 30 and 180 min. Considering all of these results, we expect that most of the particles will be effectively internalized into the cellular space after 15 to 30 min, both via endocytic clathrin-dependent and clathrin-independent pathways [85]. 

Lastly, we performed a hemolysis assay using human red blood cells and evaluated whether our hybrid UA-loaded nanoparticles could cause damage to these cells. This is very important information in terms of the potential intravenous administration of nanoformulation because hemolysis results in anemia during chemotherapy, where chemotherapy directly forces the erythrocyte membrane to break down [86]. We established that at the IC50 concentration, our nanoformulation did not induce the hemolysis of the erythrocytes. This suggests that our nanoparticles are biocompatible and without unfavorable effects on human erythrocytes, which is a very promising result in terms of further potential usage.

## 4. Materials and Methods

### 4.1. Chemical and Reagents

PLGA Resomer RG 503 H, poly(D,L-lactide-*co*-glycolide), 50:50, Mw 24,000–38,000 was acquired from Evonik, Essen, Germany. SPC 90G (soy phosphatidylcholine, Phospholipon 90G), DDAB (dimethyldioctadecylammonium (bromide salt), and DSPE-PEG 2000 (N-(carbonyl-methoxypolyethylenglycol-2000)-1,2-distearoyl-sn-glycero-3-phosphoethanolamine 2000) were purchased from Lipoid, Ludwigshafen, Germany. Ursolic acid was purchased from Wuxi Cima, China. Thiazolyl Blue Tetrazolium Bromide, 4’,6-diamidino −2-phenylindole dihydrochloride (DAPI) and Nile Red were purchased from Merck, Darmstadt, Germany. RPMI-1640 cell culture medium was purchased from Lonza, Verviers, Belgium, Fetal bovine serum, GlutaMAX (L-alanyl-L-glutamine dipeptide in 0.85% NaCl), and 100× antibiotic-antimycotic were purchased from Life Technologies (Gibco/Life Technologies, Warszawa, Poland). Dimethylsulfoxide (DMSO) was purchased from ChemPur, Piekary Slaskie, Poland. Uranyl acetate and copper mesh (400 Mesh) with formware filters and carbon shells were purchased from Agar Scientific, Essex, UK. 

### 4.2. Hybrid PLGA/Lipid Nanoparticle Preparation

**Figure 21:**
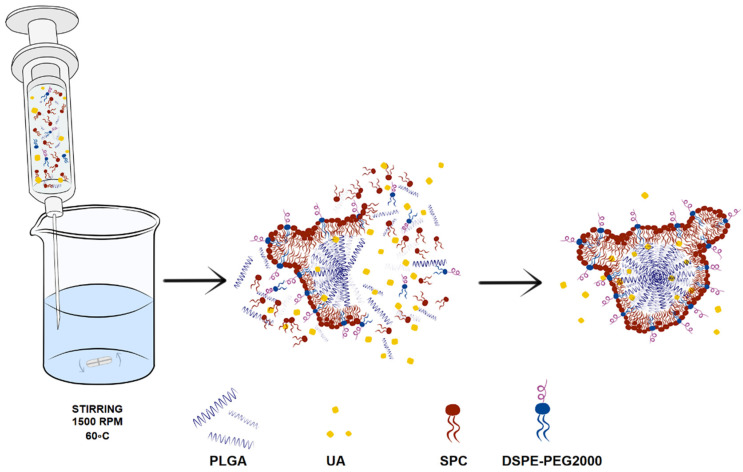
Schematic diagram representing preparation of UA-S85-PLGA-PEG 2000 hybrid nanoparticles by the nanoprecipitation method. In the first stage, PLGA, lipids, and UA are injected into Milli-Q ultrapure water under constant stirring and heating. Hybrid UA-loaded PLGA/lipid particles are formed spontaneously with UA entrapment during particle formation.

Nanoparticles were prepared by a nanoprecipitation method. Polymers, lipids, and UA were dissolved in DMSO and mixed together as an oil phase. Then, this oil phase was added to ultrapure water using a syringe, with stirring, at a temperature of 60 °C. After formation, the nanoparticles were cooled down to room temperature and separated from non-encapsulated UA and DMSO residues using gel filtration with a Sephadex G-50 Fine microcolumn. A schematic diagram of UA-loaded hybrid nanoparticle preparation is presented in Figure 21.

### 4.3. Determination of Hybrid Nanoparticles’ Size and Zeta Potential

For each experiment, size, polydispersity (PDI), and zeta potential were measured using a Malvern NanoZS dynamic light scattering system (Malvern Industries, Malvern, UK). Measurements were made in ultrapure Milli-Q water under room temperature conditions. DLS measurement graphs were made using built-in averaging software to acquire a single sample peak, made from three separate runs (*n* = 3).

### 4.4. Determination of UA Encapsulation Efficiency (EE%)

Encapsulation efficiency was determined by measuring the UA concentration in the final nanoparticle suspensions after the separation of the sample on a Sephadex G-50 Fine microcolumn. The UA concentration in the final PLGA suspensions was established using a Waters 600 HPLC system with a Phenomenex Kinetex C18 column (Phenomenex, Torrance, CA, USA) (100 cm × 2 mm). Isocratic elution was performed over 10 min using an 80:20 acetonitrile:methanol composition at a flow rate of 1 mL/min. The HPLC system was equipped with a UV detector set to 210 nm. The encapsulation efficiency was calculated using the formula:

(1) $$\frac{\mathrm{CasxD}}{\mathrm{Cbs}}\times 100\%$$

where Cas stands for the UA concentration after separation, Cbs stands for the UA concentration before separation (initial UA concentration), and D stands for the dilution of the sample during separation.

### 4.5. Hybrid Nanoparticles’ Stability Evaluation

The size, PDI, and zeta potential of loaded and unloaded UA-hybrid nanoparticles were measured immediately after preparation (day 0) and during storage at 4 °C for 1, 3, 7, 14, 28, 60, and 90 days.

### 4.6. Analysis of Loaded and Unloaded UA-Hybrid Nanoparticles by Transmission Electron Microscopy

Visualization of loaded and unloaded UA-PLGA hybrid nanoparticles was performed using a JEM F200 electron microscope (Jeol, Peabody, IN, USA). A total of 10 µL of nanoparticles suspended in ultrapure Milli-Q water was applied on a copper grid 400 mesh. After one minute, any excess of the sample was removed, and sample contrasting was performed in the presence of 2% uranyl acetate for one minute under a current of 80 kV.

### 4.7. Cell Culture

AsPC-1 (from ascites of a patient with PDAC) and BxPC-3 (primary pancreatic tumor) cells (ATCC. Manassas, VA, USA) were maintained with RPMI-1640 medium supplemented with 10% heat-inactivated fetal bovine serum (FBS), antibiotic–antimycotic mixture, and GlutaMAX solution under aseptic conditions in a Memmert ICO150 Med incubator (Memmert, Schwabach, Germany). NHDF (normal human dermal) cells (ATCC, Manassas, VA, USA) were maintained with MEM-Alpha medium supplemented with 10% heat-inactivated fetal bovine serum (FBS), antibiotic–antimycotic mixture, and GlutaMAX solution under aseptic conditions. Cultures were maintained at 37 °C in a humidified atmosphere containing 5% CO_2_.

### 4.8. MTT Cell Viability Assay

The effect of UA-loaded hybrid nanoparticles was determined using a quantitative colorimetric MTT assay adapted from Mosmann [87]. Cells were seeded in 96-well plates (4500 cells per well) in an appropriate complete cell culture medium for 24 h. Cells were treated with UA encapsulated in PLGA hybrid nanoparticles, UA dissolved in DMSO in the range of 2.5–80 µM (an equivalent volume of DMSO was used as a negative control, maximal concentration was 0.18% *v*/*v*), or control unloaded hybrid nanoparticles for 72 h. The medium containing the tested formulations was removed, MTT solution (working solution: stock 0.5 mg/mL was 10-fold diluted in medium) was added to the wells, and the plates were incubated for a further 3 h. Subsequently, the MTT solution was replaced with DMSO (50 µL/well) to dissolve the purple formazan crystals. Absorbance was measured at 560 nm, with a reference wavelength of 670 nm, on an Asys UVM 340 microplate reader (Cambridge, UK). Results were expressed as the percentage of surviving cells, with respect to the control (the untreated cells), calculated as:

Cell viability (%) = (AT/AC) × 100,(2)

where:

AT = absorbance of the treatment well (treated cells);
AC = absorbance of the control well (untreated cells).

### 4.9. Cellular Uptake

Cellular uptake of Nile Red PLGA hybrid nanoparticles by AsPC-1 and BxPC-3 cells was assessed by fluorescence microscopy. Nile Red was encapsulated in nanoparticles using exactly the same procedure used for UA. Cancer cells were seeded onto glass cover slides placed in 12-well culture plates. After 24 h incubation, the cell culture medium was replaced with a medium containing Nile-Red-loaded hybrid PLGA nanoparticles. The cells were incubated at 37 °C for 15 and 120 min. Subsequently, cells were washed three times with PBS (37 °C) to remove excess nanoparticles and fixed for 20 min in 4% paraformaldehyde, washed with phosphate-buffer saline (PBS), and stained with DAPI. Slides were analyzed using a Leica TCS SP8 confocal microscope (Leica Microsystems, Mannheim, Germany) with an HC PL APO CS2 63×/1.40 oil objective. To excite Nile Red and DAPI, a fluorescent probe that forms a complex by fixing to DNA and 561 and 405 nm lasers (Leica Microsystems, Mannheim, Germany), respectively, were used.

### 4.10. Determination of Hemolysis of Human Erythrocytes

Hemolysis was evaluated according to the procedure of Jaromin et al. [86]. The study protocol was approved by the Bioethics Commission at the Lower Silesian Medical Chamber (1/PNHAB/2018). UA-S85-PLGA-PEG 2000 nanoparticles were added in a volume corresponding to a final concentration of 12 µM in the sample and incubated with freshly isolated human erythrocytes in PBS (30 min, 37 °C). The released hemoglobin was measured after centrifugation at 540 nm. Negative (erythrocytes in PBS buffer) and positive (erythrocytes in distilled water) controls were also determined.

### 4.11. Statistical Analysis

Data are presented as mean ± standard deviation. Statistical analyses were made using GraphPad Prism software (Version 7, GraphPad Software, San Diego, CA, USA) with a one-way ANOVA (Prism 7 for Windows) and Tukey’s multiple comparisons test, with a 95% confidence interval.

## 5. Conclusions

We designed, prepared, and evaluated new smart UA-loaded nanoparticles with improved parameters of stability and retained proper morphological values and cytotoxicity potential that are similar to our previous PLGA-based nanoformulations of this compound. We propose a nanocarrier system for potential drug delivery that combines liposomal and polymeric aspects. This system is suitable for surface modifications to achieve desirable pharmacokinetics, enabling active targeting or application in gene therapy, and can be administered via different routes by modifying the composition of the used lipid bilayer. Our second-generation hybrid nanoparticles could lead to the development of a third generation, where, besides PEG modification, particles could be modified with targeting agents to enhance their anticancer properties even more.

## Figures and Tables

**Table 1:** UA-loaded hybrid nanoparticle compositions with respective abbreviations.

Sample Abbreviation	Sample Composition
UA-PLGA	Ursolic-acid-loaded PLGA nanoparticles
UA-S100-PLGA	Ursolic-acid-loaded PLGA nanoparticles with lipid coats composed of pure SPC 90G
UA-S95-PLGA-PEG 2000	Ursolic-acid-loaded PLGA nanoparticles with lipid coats composed of SPC 90 and DSPE-PEG 2000 in molar ratio 95:5
UA-S85-PLGA-PEG 2000	Ursolic-acid-loaded PLGA nanoparticles with lipid coats composed of SPC 90 and DSPE-PEG 2000 in molar ratio 85:15

**Table 2:** Nanoparticles’ characterization: size, PDI, and zeta potential values; nd—not determined.

Sample	PLGA	UA-PLGA	S100-PLGA	UA-S100-PLGA	S95-PLGA-PEG 2000	UA-S95-PLGA-PEG 2000	S85-PLGA-PEG 2000	UA-S85-PLGA-PEG 2000
Size [nm]	110.6 ± 0.2	206.8 ± 3	167.2 ± 2.8	638.5 ± 445.7	146.3 ± 1	167.2 ± 2.7	130.3 ± 2.4	145.1 ± 2.6
PDI	0.05 ± 0.01	0.26 ± 0.1	0.09 ± 0.01	0.63 ± 0.2	0.07 ± 0.01	0.08 ± 0.02	0.09± 0.02	0.1 ± 0.01
Zeta potential [mv]	−34.3 ± 1.1	−30.4 ± 0.3	−31.7 ± 0.7	nd	−39.8 ± 0.9	−32.5 ± 1	−34.6 ± 3	−42 ± 1.2

**Table 3:** Calculated IC50 values for encapsulated and non-encapsulated UA after 72 h of incubation in two PDAC cell lines: AsPC-1 and BxPC-3. NHDF was tested as well as normal cell controls.

Sample	AsPC-1	BxPC-3	NHDF
UA-DMSO	11.3 ± 1.7	17.9 ± 5.3	12.9 ± 3.9
UA-S85-PLGA-PEG 2000	8.6 ± 0.5	12.0 ± 5.7	15.4 ± 2.9
S85-PLGA-PEG 2000	Non-toxic	Non-toxic	Non-toxic
DMSO	Non-toxic	Non-toxic	Non-toxic

## Data Availability

The data are stored by the corresponding author and available upon request.
